# Diffusible Tumor Necrosis Factor-Alpha (TNF-α) Promotes Noise-Induced Parvalbumin-Positive (PV+) Neuron Loss and Auditory Processing Impairments

**DOI:** 10.3389/fnins.2020.573047

**Published:** 2020-10-12

**Authors:** Di Deng, Weihua Wang, Shaowen Bao

**Affiliations:** Department of Physiology, College of Medicine, University of Arizona, Tucson, AZ, United States

**Keywords:** hearing loss, TNF-α, parvalbumin-positive neuron, microglia, auditory processing, tinnitus

## Abstract

Neuroinflammation has been implicated in noise-induced auditory processing disorder and tinnitus. Certain non-auditory neurological disorders can also increase the levels of proinflammatory cytokines in the brain. To investigate the impact of increased brain proinflammatory cytokine levels on the central auditory pathway, we infused recombinant TNF-α into the right lateral cerebral ventricle, and examined auditory processing and cytoarchitecture of the auditory cortex. Microglial deramification was observed in the auditory cortex of mice that had received both TNF-α infusion and exposure to an 86-dB noise, but not in mice that had received either TNF-α infusion or noise exposure alone. In addition, we observed reduced cortical PV+ neuron density and impaired performances in gap detection and prepulse inhibition (PPI) only in mice that received both TNF-α infusion and the noise exposure. These results suggest that disease-related increase in brain proinflammatory cytokine release could be a risk factor for noise-induced auditory processing disorder and tinnitus.

## Introduction

Hearing loss often results in impairment in gap detection (Fitzgibbons and Wightman, 1982), which is considered a sign of temporal processing deficit and possibly tinnitus (Turner et al., 2006; Fournier and Hebert, 2013). In animal models, gap detection is often examined with the gap prepulse-induced inhibition of the startle response (Turner et al., 2006). Impairment in this behavioral paradigm is correlated with several pathological changes in the auditory pathway, such as enhanced excitatory synapses, weakened inhibitory synapses and increased neuronal membrane excitability (Abbott et al., 1999; Milbrandt et al., 2000; Kotak et al., 2005; Yang et al., 2011; Browne et al., 2012; Llano et al., 2012; Yang et al., 2012; Li et al., 2013, 2015; Sturm et al., 2017). A potential modulator of these synaptic and membrane properties is the neuroinflammatory process and the proinflammatory cytokine TNF-α (Bellinger et al., 1993; Stellwagen et al., 2005; Stellwagen and Malenka, 2006; Di Filippo et al., 2008; Steinmetz and Turrigiano, 2010). Hearing loss causes inflammatory responses along the central auditory pathway, and an increase in the expression of several proinflammatory cytokines (Fuentes-Santamaria et al., 2014; Baizer et al., 2015; Fuentes-Santamaria et al., 2017; Wang et al., 2019). Among them, TNF-α has been shown to play a central role in organizing the inflammatory response in the brain. Its expression is rapidly upregulated following noise-induced hearing loss in mice, followed by microglial activation and increased expression of other proinflammatory cytokines (Wang et al., 2019). Blocking noise-induced increase of TNF-α expression prevented both synaptic imbalance and behavioral impairment in gap detection in noise exposed mice (Wang et al., 2019). In the same study, blocking TNF-α expression also prevented behavioral evidence of tinnitus assessed with an operant perceptual task (Wang et al., 2019). In humans, polymorphisms of several inflammatory cytokines, including TNF-α, are associated with the risk of noise-related tinnitus (Doi et al., 2015; Marchiori et al., 2018; Marchiori et al., 2019), a central auditory disorder that is often comorbid with impairment in gap detection (Fournier and Hebert, 2013; Mehdizade Gilani et al., 2013). The level of interleukin 10, an anti-inflammatory cytokine also known as human cytokine synthesis inhibitory factor, was lower in people with tinnitus, but not in people who had hearing loss without tinnitus (Haider et al., 2020).

In addition to hearing loss, several non-auditory pathologies and health conditions that are known to be associated with neuroinflammation can also increase the risk of tinnitus and auditory processing disorders. For example, traumatic brain injury (TBI), which causes neuroinflammatory responses (Simon et al., 2017), increases the risk of tinnitus and central auditory processing disorders. The incidence of tinnitus is higher in military personnel with TBI compared to those without TBI (Lew et al., 2007). Blast-exposed military service members exhibit a threefold increase in abnormalities on tests of central auditory function and speech in noise, despite normal peripheral hearing sensitivity (Gallun et al., 2012; Saunders et al., 2015). Repetitive head impacts even without concussion can result in central auditory processing disorders (Malloy and Bellis, 2013). Stress, which causes neuroinflammation in many brain regions (Grippo and Scotti, 2013; Calcia et al., 2016), is also a risk factor and modulator for tinnitus (Mazurek et al., 2015). Depression and pain, which are not only mere comorbidities, but also risk factors of tinnitus (Wright and Gullickson, 1996; Isaacson et al., 2003; Langguth et al., 2011; Popeo et al., 2011; Walker et al., 2014; Durai and Searchfield, 2016), are also associated with neuroinflammation (DeLeo and Yezierski, 2001; Kiguchi et al., 2012; Ellis and Bennett, 2013). All these diverse non-auditory risk factors could potentially increase proinflammatory cytokine concentration in the cerebrospinal fluid (Levine et al., 1999; Bianchi et al., 2007; Juengst et al., 2014; Lerman et al., 2016), providing a diffusible signal to influence the function of the central auditory system.

In this study, we examined whether intracerebroventricular administration of the proinflammatory cytokine TNF-α increases the risk of auditory impairment in mice with and without hearing loss. We found that exposure to 86 dB SPL 8-kHz tone resulted in approximately a 20-dB threshold increase but no microglial deramification in the auditory cortex. Intracerebroventricular infusion of recombinant TNF-α also failed to induce microglial deramification in the auditory cortex. When combined, noise exposure and TNF-α infusion synergistically activated microglia in the auditory cortex signified by significant microglial deramification, reduced PV+ neuron density, and impaired gap detection and prepulse inhibition (PPI). These results suggest that increase proinflammatory cytokine in the central nervous system can increase the risk of noise-induced tinnitus and/or temporal processing deficits.

## Materials and Methods

### Animals

Animal experiments were performed in accordance with the guidelines of the National Institutes of Health, as approved by the University of Arizona Institutional Animal Care and Use Committees. Male C57BL/6 mice (2–3 months, 20–25 g; The Jackson Laboratory No. 000664) were housed in groups of five per cage with a 12 h light/dark cycle.

### Osmotic Pump Implantation

Mice were anesthetized with isoflurane (∼1% in a gas mixture) in stereotaxic apparatus. After anesthesia, an ALZET mini-osmotic pump (Model 1007D, ALZET, DURECT Co., Cupertino, CA, United States) was implanted subcutaneously in the back of the animal, slightly posterior to the scapulae according to the ALZET instruction. Polyethylene tubing, leading from the ALZET osmotic pump, was implanted in the right lateral ventricle (AP: −0.5 mm; ML: −1 mm; DV: 2 mm) using the ALZET Brain Infusion Kit (Kit3, ALZET) and adhesive luting cement (C&B Metabond Quick Adhesive Cement System, Parkell, Inc., NY, United States) (Altura et al., 1998). ALZET Model 1007D osmotic pump delivered 0.5 μL per hour for 7 days. Prior to implantation, the osmotic pumps were filled with 100 μL sterile saline containing recombinant mouse TNF-α protein (Cat. 410-MT-050, RND Inc., MN, United States) at a dose that delivers 1 μg/day/kg body weight.

### Noise Exposure

The day after pump implanting, mice were anesthetized with isoflurane (∼1% in a gas mixture) and maintained at 36.5°C with a homeothermic heating pad (Harvard Apparatus, Holliston, MA, United States). Unilateral Noise-induced hearing loss was induced by playing a continuous 8-kHz tone at 86 dB SPL through a custom-made piezoelectric earphone speaker to the left ear for 2 h. The right ear was protected with sound attenuating clay. The sound level was measured with a Bruel and Kjaer 4135 condenser microphone (Narum, Denmark).

### Auditory Brainstem Response (ABR) Recording

Under isoflurane anesthesia, auditory brainstem responses (ABRs) were recorded to assess hearing thresholds before and after noise exposure. ABR signals were acquired using the BioSigRP software on a TDT RX5 Sys3 recording rig. Tone pips (3-ms full-cycle sine waves of various frequencies, with 0.25-ms Cos2-gated rise/fall time, at 5-dB intensity steps from 0 to 70 dB) were delivered to a single ear through a cannulated speaker at a rate of 19 times per second. The speaker was calibrated to have <3% harmonic distortion and flat output in the entire frequency range (Tucker-Davis Technologies SigCal32). Five hundred recordings were averaged for each frequency intensity pair. ABRs were visually identified by an experienced experimenter who was “blind” to the animals’ group identities. The lowest sound level that evoked discernable ABRs was determined at each testing frequency and recorded as the ABR threshold for that frequency.

### Gap Detection Test and PPI Test

The behavioral test and experimental procedures were the same as previously reported (Wang et al., 2019). Briefly, a mouse was caged in a plastic container with a mesh lid. The container was placed on a piezoelectric sensor in a sound attenuation chamber. Sounds were played through an open field speaker (FOSTEX FT17H) fixed above the container. The gap detection task measured the acoustic startle response elicited by a brief white noise pulse and its suppression by a preceding silent gap embedded in a continuous background sound. Each trial started with a carrier pure tone (frequency pseudorandomly selected from 5, 7, 10, 14, 20, and 28 kHz, all at 75 dB SPL) played for a duration of 10–20 s. In uncued trials, the carrier tone was followed by a startle stimulus of a 50-ms white noise burst at 102 dB SPL. In cued trials, a 50-ms silent gap in the background sound was introduced starting 100 ms before the onset of the loud noise burst. In each testing session, the animal underwent a total of 500 trials (50% cued and 50% uncued). After each session, we calculated the startle response ratio, which is defined as the average startle amplitude in the silent gap-cued trials divided by the average amplitude in the uncued trials. A lower startle response ratio indicates better detection of the silent gap. A startle response ratio of 1 suggests that the animal failed to detect the silent gap.

To assess an animal’s ability to hear a sound and perform an auditory task, animals underwent the PPI test before and after noise exposure. The test apparatus for the PPI task was identical to that of the gap detection. However, the test differed in that carrier tone was absent and a white noise burst was cued by a 50-ms pure tone pulse (frequency pseudorandomly selected from 5, 7, 10, 14, 20, and 28 kHz, all at 75 dB SPL). In short, the PPI task tests an animal’s ability to detect a pure tone pulse in silence, while the gap detection task measures an animal’s ability to detect a silent gap in a continuous pure tone. Gap detection and PPI performances were stabilized over two daily sessions each before data were collected.

### Immunofluorescence Staining and Image Analysis

Ten days after noise exposure, mice were transcardially perfused under deep anesthesia with ice-cold phosphate-buffered saline (PBS) followed by 4% paraformaldehyde. Brains were removed and fixed in the same fixative overnight at 4°C, equilibrated in 30% sucrose. Coronal sections (16 μm in thickness) were cut by Vibrating blade microtome (Leica VT1000 S) and collected on gelatinized glass slides. Sections were systematically and evenly sampled from the rostral-caudal extend of AI. After air drying, sections were washed in PBS and penetrated with 0.3% Triton-X at room temperature for 10 min. The tissue samples were blocked with Dako Serum-free blocking buffer (Dako) and incubated with primary antibodies (parvalbumin polyclonal antibody, PA1-933, Invitrogen; ionized calcium binding adaptor molecule 1 (IBA1) polyclonal antibody, 019-19741, FUJIFILM Inc.) overnight at 4°C. The secondary antibodies conjugated with Alexa Fluor 488 (anti-PV antibody) or 568 (anti-IBA1 antibody; Invitrogen) were incubated for 1 h at room temperature to enable fluorescent detection. After rinsing with PBS, the sections were mounted with fluorescence mounting medium (Dako) and viewed under the Olympus BX40 microscope with a digital microscope camera (C11440; Hamamatsu). Immunofluorescent images were acquired and analyzed by experienced experimenters who were blind to the sample groups. During image acquisition, the camera and software setting were fixed across all groups for each experimental condition. All the images were taken on the same day using the same parameters. Analysis of the PV and IBA1 staining was performed with the software ImageJ. PV+ neuron density was counted manually and corrected for the area size of AI (Jensen, 2013; Wang et al., 2018). The whole cell body and the soma of each microglial cell were separately outlined manually. Then the image was thresholded, and the suprathreshold pixels were counted in ImageJ for the whole cell body and the soma to derive the soma-to-whole body ratio, which was used to measure microglial activation (Hovens et al., 2015; Wang et al., 2018).

### Statistical Analysis

Statistical analyses were conducted using GraphPad Prism 7.0. Gap detection, pre-pulse inhibition test and ABR test were analyzed by two-way analysis of variance (ANOVA) for different sound frequency and different treatment group. When statistical significance was reached at *p* < 0.05, *post hoc* analyses were performed using Bonferroni’s test. All data are expressed as mean ± standard error of the mean (SEM), and *p* < 0.05 was considered statistically significant.

## Results

### Exposure to an 86-dB and 8-kHz Tone Resulted in Mild Hearing Loss

**FIGURE 1:**
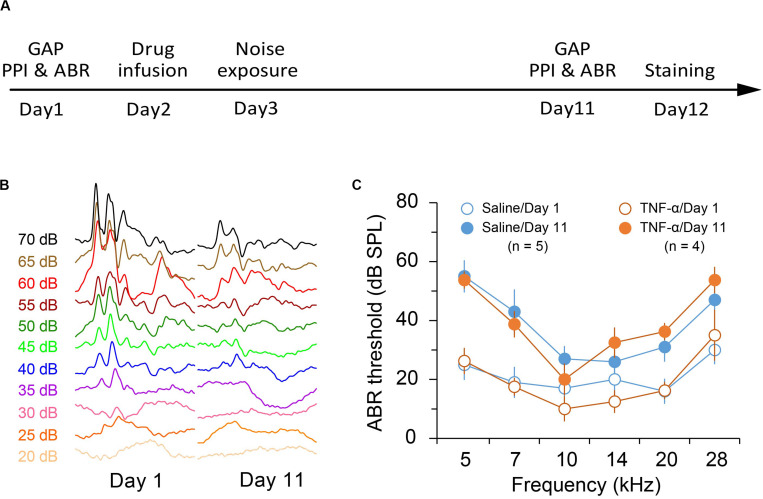
Experimental design and noise-induced hearing loss. **(A)** Experimental timeline. **(B)** Representative traces of auditory brainstem responses in saline-infused mice before and after noise exposure. **(C)** Auditory brainstem response (ABR) threshold in saline- and TNF-α-infused mice before and after noise exposure. Data are presented as mean ± SEM.

Previous reports indicated that exposure to an 8-kHz tone at 112 to 114 dB SPL for 2 h resulted in a moderate threshold increase of 40–50 dB SPL (Miyakawa et al., 2019; Wang et al., 2019). In this study we aimed to produce a mild hearing loss. We found that exposure to an 86-dB and 8-kHz tone for two hours reliably caused a threshold increase of approximately 20–30 dB SPL in the exposed ear (TNF-α × Noise × Frequency repeated measures ANOVA, effect of noise exposure, *F*_1_,_35_ = 65.143, *p* < 0.001; Figure 1C). TNF-α infusion had no effect on noise-induced threshold shift (effect of TNF-α, *F*_1_,_7_ = 0.007, *p* = 0.934; TNF-α × Noise interaction, *F*_1_,_35_ = 0.325, *p* = 0.587; Figure 1).

### TNF-α Infusion Increased Vulnerability to Noise-Induced Impairments in Gap Detection and PPI

**FIGURE 2:**
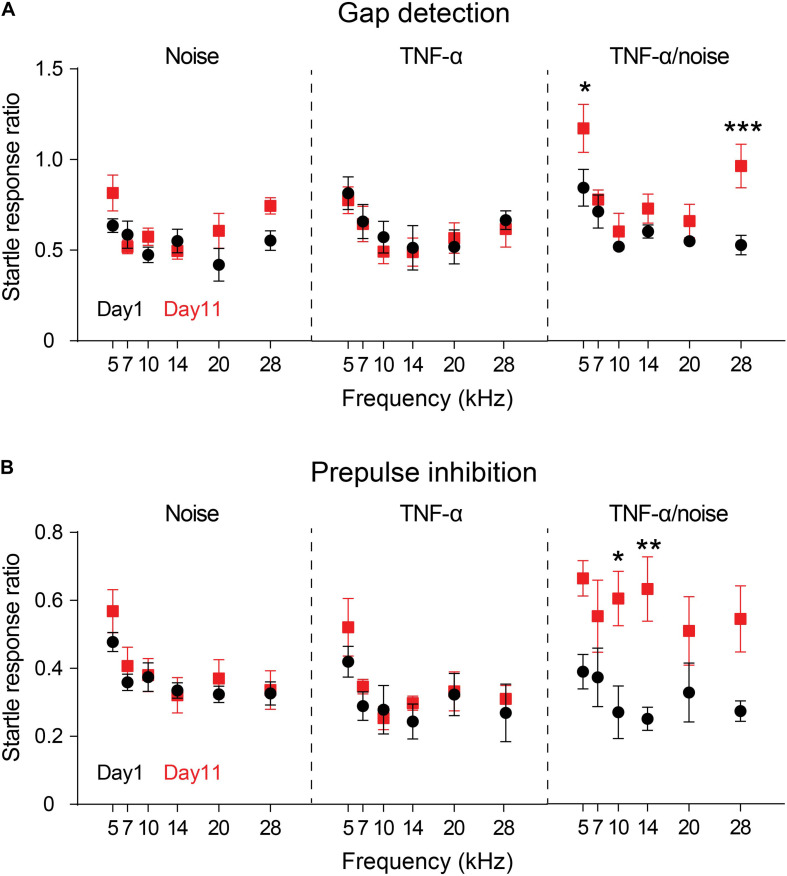
TNF-α infusion increases vulnerability to noise-induced impairments in gap detection and PPI. Animals’ performances in gap detection **(A)** and prepulse inhibition **(B)** before (Day 1) and after (Day 11) noise exposure (Noise), TNF-α infusion (TNF-α) and combined TNF-α infusion and noise exposure (TNF-α/Noise). Data are presented as mean ± SEM. * indicates *p* < 0.05, **p < 0.01, and ***p < 0.005. Both gap detection and prepulse inhibition were impaired in mice that had received combined TNF-α infusion and noise exposure.

Previous reports showed that exposure to a 112-dB and 8-kHz tone for two hours resulted in impairment in gap detection but not PPI (Miyakawa et al., 2019; Wang et al., 2019). Exposure to an 86-dB tone in the present study resulted in no significant impairment in either gap detection or PPI (Noise × Frequency repeated measures ANOVA, effect of noise exposure on gap detection, *F*_1_,_4_ = 3.415, *p* = 0.14; effect of noise exposure on PPI, *F*_1_,_4_ = 0.4896, *p* = 0.52; *n* = 5 mice; Figures 2A,B). Infusion of TNF-α alone did not alter the performances in gap detection or PPI in normal hearing animals (TNF-α × Frequency repeated measures ANOVA, effect of TNF-α on gap detection, *F*_1_,_4_ = 2.739, *p* = 0.17; effect of TNF-α on PPI, *F*_1_,_4_ = 4.383, *p* = 0.10; *n* = 5 mice). However, the animals that had received TNF-α infusion and noise exposure exhibited impairments in auditory processing, as their performances in both gap detection and PPI were significantly worsened (gap detection, *F*_1_,_4_ = 9.71, *p* < 0.05; PPI, *F*_1_,_4_ = 8.076, *p* < 0.05; *n* = 5 mice; Figures 2A,B). The impairments in gap detection were found at 5 and 28 kHz (Bonferroni’s test, *p* = 0.048 at 5 kHz and *p* = 0.0035 at 28 kHz), whereas the impairments in PPI were at 10 and 14 kHz (*p* = 0.026 at 10 kHz and *p* = 0.0079 at 14 kHz).

To examine the effect of TNF-α infusion on the vulnerability of noise-induced auditory processing disorder, we compared the performances of the Noise group and the TNF-α + noise group. A TNF-α × Noise × Frequency 3-way repeated measures ANOVA revealed a significant effect of TNF-α infusion (*F*_1_,_8_ = 20.306, *p* = 0.002), suggesting that animals that had received TNF-α infusion were more vulnerabe to noise-induced auditory processing disorder.

### TNF-α Infusion and Noise Exposure Synergistically Induced Microglial Deramification

**FIGURE 3:**
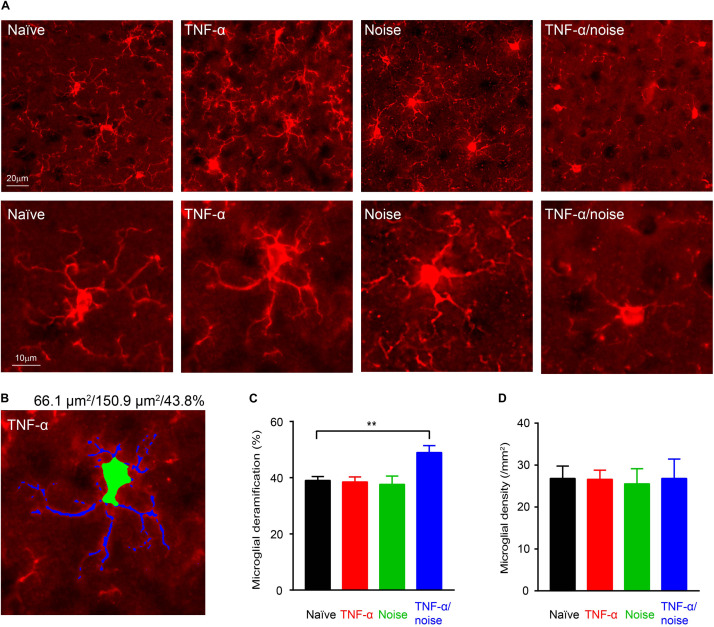
TNF-α infusion and noise exposure synergistically induce microglial deramification. **(A)** Representative images of microglia stained with an IBA1 antibody. Bottom row shows enlarged images of individual microglial cells. **(B)** An example microglial cell with its soma labeled green and dendrites labeled blue. Given on top are the sizes of its soma and whole body, and the soma-to-whole body ratio. **(C)** Microglial deramification index was enhanced only in mice that had received both TNF-α infusion and noise exposure. **(D)** Microglial density was not altered by TNF-α infusion and/or noise exposure. All data are presented as mean ± SEM. ** indicates *p* < 0.01.

Exposure to a 112-dB tone were shown to activate cortical microglia as indicated by their morphological deramification (Wang et al., 2019). By contrast, we did not observe microglial deramification in the auditory cortex in mice that had been exposed to an 86-dB tone (Figures 3A–C). Although TNF-α infusion alone did not lead to microglial deramification in the auditory cortex, it resulted in cortical microglial deramification in mice that had subsequently been exposed to a 86-dB tone (*n* = 48 cells/4 mice for the Naïve group, 48 cells/4 mice for the TNF-α group, 30 cells/4 mice for the Noise group, and 24 cells/3 mice for the TNF-α/Noise group; one-way ANOVA, *F*_3_,_146_ = 6.057, *p* < 0.01; *post hoc* Bonferroni’s test, Naïve vs TNF-α/noise, *p* < 0.01; Figure 3C). The density of cortical microglia was not different among the four groups (*n* = 7 sections/4 mice for the Naïve group, 7 sections/4 mice for the TNF-α group, 3 sections/3 mice for the Noise group, 3 sections/3 mice for the TNF-α/Noise group; one-way ANOVA, *F*_3_,_16_ = 0.1997, *p* = 0.89; Figure 3D).

### TNF-α Infusion and Noise Exposure Synergistically Induced PV+ Neuron Loss

**FIGURE 4:**
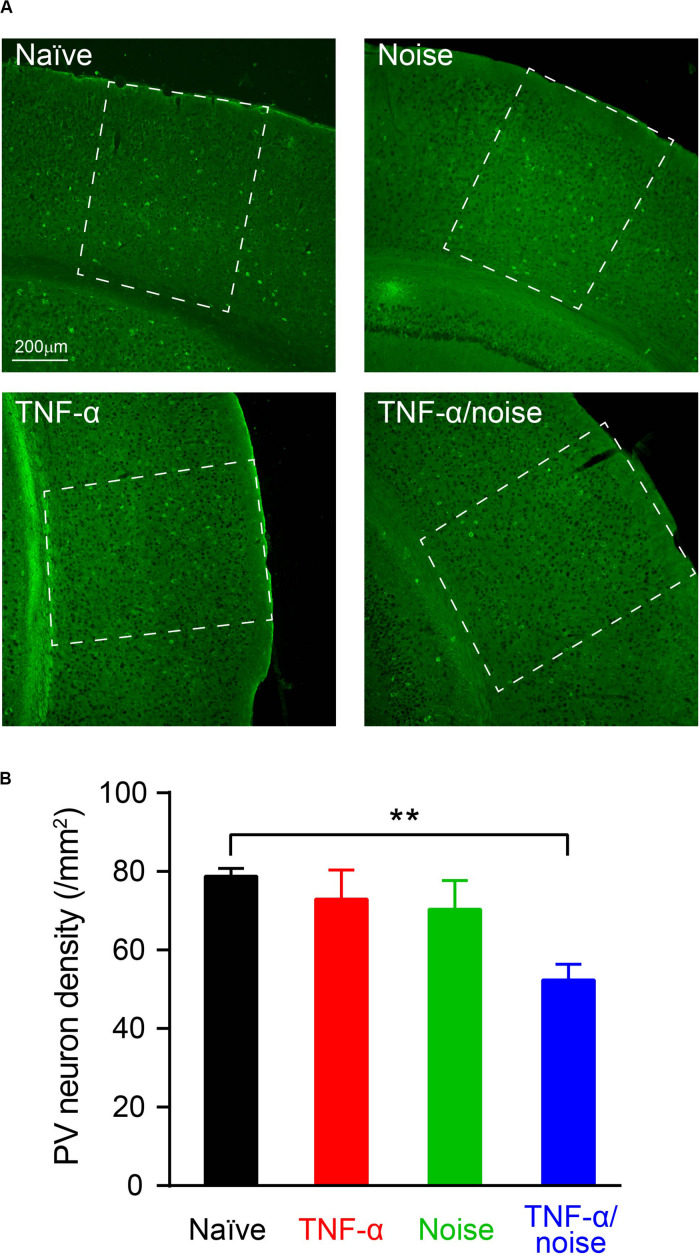
TNF-α infusion and noise exposure synergistically induced PV+ neuron loss. **(A)** Representative images of PV+ neurons stained with a parvalbumin antibody. **(B)** PV+ neuron density was reduced only in mice that had received both noise exposure and TNF-α infusion. All data are presented as mean ± SEM. ** indicates *p* < 0.01.

Cortical PV+ neurons can regulate excitation/inhibition balance (Ferguson and Gao, 2018), and encode dynamic stimulus features such as gap in sounds (Weible et al., 2014; Keller et al., 2018). Reduced PV+ neuron density was observed in the hippocampus following blast-induced neuroinflammatory responses (Wang et al., 2018). We quantified PV+ neuron density to determine whether TNF-α infusion and/or noise exposure caused PV+ neuron loss (Figure 4A). Compared to the naïve group, only TNF-α/Noise group showed a reduction in PV+ neuron density (*n* = 13 sections/4 mice for the Naïve group, 8 sections/4 mice for the TNF-α group, 10 sections/4 mice for the Noise group, 10 sections/4 mice for the TNF-α/Noise group; one-way ANOVA, *F*_3_,_37_ = 5.075, *p* < 0.01; *post hoc* Bonferroni’s test, *p* < 0.01, Naïve vs TNF-α/Noise; Figure 4B).

## Discussion

In this study, we showed that intraventricular infusion of recombinant TNF-α facilitates noise-induced neuroinflammatory responses and PV+ neuron loss in the auditory cortex. These cellular pathologies are paralleled by behavioral impairments in gap detection and PPI. Although the dose of TNF-α infusion in the present study is in the range to produce behavioral symptoms of hyperalgesia and depressive behaviors (Ignatowski et al., 1999; Bluthe et al., 2000), the infusion alone did not cause cortical microglial deramification or impairment in gap detection or PPI. Instead, it made the auditory system more vulnerable to noise-induced neuroinflammatory responses, cell loss and behavioral impairments. These results suggest a synergistic interaction between TNF-α and noise exposure in auditory processing disorder and tinnitus. Furthermore, many neuroinflammation-related disorders can increase proinflammatory cytokine levels in the cerebrospinal fluid, which, as our results suggest, could increase the risk of auditory processing disorders and tinnitus.

Exposure to a 8-kHz tone at 124 dB SPL for 2 h has been shown to cause a 50-dB ABR threshold increase, microglial deramification and gap detection impairment (Wang et al., 2019). In this study, exposed to a tone of the same frequency for the same duration at a level of 86 dB SPL resulted in a 30-dB threshold increase, but no microglial deramification or gap detection impairments. The different anesthetics used in the two studies (ketamine/xylazine vs isoflurane) might have also contributed to observed differences in hearing (Kim et al., 2005). There seems to be a threshold in the level of noise exposure or hearing loss that can lead to microglial deramification and gap detection impairments. In the present study, this threshold was lowered by intracerebroventricular infusion of TNF-α, a procedure mimicking the release of proinflammatory cytokine in neuroinflammatory disorders (Levine et al., 1999; Bianchi et al., 2007; Juengst et al., 2014; Lerman et al., 2016).

It is interesting to note that in the gap detection test, mice in the Noise group showed increased startle response ratio at 5 and 28 kHz, a pattern similar to that of the TNF-α + noise group (Figure 1). Although the increase was not statistically significant due to the small sample size, an increase in the sample size could reveal a small but significant impairment in gap detection by the exposure to an 86-dB noise. Nevertheless, the significant difference that we have observed between the Noise and the TNF-α + noise groups supports our conclusion that TNF-α infusion increased the vulnerability of noise-induced auditory processing disorder.

TNF-α has a rapid turnover rate in the brain and could be cleared out within four hours (Biesmans et al., 2015). Thus, the slowly infused TNF-α in the present study likely was continuously metabolized and removed from the brain. We did not measure the steady-state TNF-α concentrations in the cerebrospinal fluid or in auditory brain areas during the seven days of infusion. It is possible that the TNF-α level reached in the auditory cortex was too low to activate microglial and cause impairment in gap detection by itself. Rapid infusion of TNF-α directly into the auditory cortex was shown to result in impairment in gap detection without noise exposure (Wang et al., 2019). Increased proinflammatory cytokine levels in the cerebrospinal fluid were observed in pain, depression, stress and TBI (Levine et al., 1999; Bianchi et al., 2007; Juengst et al., 2014; Lerman et al., 2016). The TNF-α infusion dose in the present study is in the range that had been shown to produce hyperalgesia and depressive behaviors in rodent models (Ignatowski et al., 1999; Bluthe et al., 2000). Thus, our findings are likely relevant in understanding how neuroinflammation-related non-auditory disorders increase the risk of auditory impairments.

Microglia can be activated by TNF-α and inhibited by neurotrophins (Neumann et al., 1998; Kuno et al., 2005). Noise-induced hearing loss likely reduces sensory driven activity and the activity-dependent release of neurotrophins, leading to activation of microglia. Thus, TNF-α infusion and noise exposure could synergistically activate microglia through separate signaling pathways.

Noise exposure has been shown to cause neuron death in the central auditory pathway, including the auditory cortex (Basta et al., 2005; Groschel et al., 2010; Sekiya et al., 2012). Noise-induced neuroinflammatory responses in the central auditory pathway (Fuentes-Santamaria et al., 2014; Baizer et al., 2015; Fuentes-Santamaria et al., 2017; Wang et al., 2019) could potentially mediate neuronal death through apoptosis (Talley et al., 1995), necroptosis (Liu et al., 2014), glutamate excitotoxicity (Olmos and Llado, 2014), and microglial phagocytosis (Brown and Neher, 2014). Indeed, neuroinflammatory responses were shown to be involved in blast-induced PV+ neuron loss (Wang et al., 2018). In the present study, PV+ neuron loss and microglial activation occurred in the same experimental group, supporting a role of neuroinflammation in noise-induced PV+ neuron loss.

PV+ neurons account for about 40% of cortical inhibitory neurons (Xu et al., 2010). PV+ neuron loss contributes to noise-induced reduction of cortical neuronal inhibition, which has been implicated in impairments in gap detection (Miyakawa et al., 2019; Wang et al., 2019). PV+ neurons are involved in diverse cortical functions, such as sharpening response timing, improving signal-to-noise ratio, controlling neuronal gain, and gating the critical period of developmental plasticity (Hensch and Stryker, 2004; Gabernet et al., 2005; Sohal et al., 2009; Atallah et al., 2012; Lee et al., 2012; Hamilton et al., 2013). They respond to the onset of gap-in-noise more strongly than non-PV neurons (Keller et al., 2018). Suppressing cortical PV+ neuron activity alters gap detection performance (Weible et al., 2014). Furthermore, PV-deficient mice are impaired in PPI (Brown et al., 2015). In the present study, PV+ neuron loss accompanied by impairment in gap detection and PPI, supporting the notion that PV+ neuron loss contributes to impairments in gap detection as a model of auditory processing disorder and tinnitus.

The results of the present study add to converging evidence that the central auditory pathway can release proinflammatory cytokines under pathological conditions such as noise trauma, and proinflammatory cytokines can in turn increase the risk of auditory processing disorder and tinnitus. Cytokines are diffusible in the nerve tissue, and can even cross the blood-brain barrier (Banks et al., 1995), providing a mechanism for the interaction between auditory and non-auditory systems in inflammation-related pathologies, such as pain, depression and dementia (Wright and Gullickson, 1996; Isaacson et al., 2003; Langguth et al., 2011; Popeo et al., 2011; Walker et al., 2014; Durai and Searchfield, 2016; Suhnan et al., 2017; Thomson et al., 2017). Incorporating inflammation in hearing studies could provide new insight into these hearing-related disorders. Recognizing neuroinflammation as a risk factor may also help to improve the care for and protection against hearing disorders.

## Data Availability Statement

The raw data supporting the conclusions of this article will be made available by the authors, without undue reservation.

## Ethics Statement

The animal study was reviewed and approved by University of Arizona Institutional Animal Care and Use Committees.

## Author Contributions

SB conceived the research plan. DD and WW carried out the experiments and analyzed the results. All authors wrote the manuscript.

## Conflict of Interest

The authors declare that the research was conducted in the absence of any commercial or financial relationships that could be construed as a potential conflict of interest.
